# Safety and efficacy of ribociclib plus letrozole in patients with HR+, HER2– advanced breast cancer: Results from the Spanish sub-population of the phase 3b CompLEEment-1 trial

**DOI:** 10.1016/j.breast.2022.09.006

**Published:** 2022-09-28

**Authors:** Javier Salvador Bofill, Fernando Moreno Anton, Cesar Augusto Rodriguez Sanchez, Elena Galve Calvo, Cristina Hernando Melia, Eva Maria Ciruelos Gil, Maria Vidal, Begoña Jiménez-Rodriguez, Luis De la Cruz Merino, Noelia Martínez Jañez, Rafael Villanueva Vazquez, Ruben de Toro Salas, Antonio Anton Torres, Isabel Manuela Alvarez Lopez, Joaquin Gavila Gregori, Vanesa Quiroga Garcia, Elena Vicente Rubio, Juan De la Haba-Rodriguez, Santiago Gonzalez-Santiago, Nieves Diaz Fernandez, Agusti Barnadas Molins, Blanca Cantos Sanchez de Ibargüen, Juan Ignacio Delgado Mingorance, Meritxell Bellet Ezquerra, Sonia de Casa, Asuncion Gimeno, Miguel Martin

**Affiliations:** a Hospital Universitario Virgen del Rocío, Instituto de Biomedicina de Sevilla (IBIS), Sevilla, Spain; b Hospital Clínico San Carlos, Madrid, Spain; c Departamento de Oncología Médica, Hospital Universitario de Salamanca-IBSAL, Salamanca, Spain; d Hospital Universitario de Basurto, Bilbao, Spain; e Servicio de Oncología, Hospital Clínico Universitario de Valencia e Instituto de Investigación Sanitaria INCLIVA, Valencia, Spain; f Hospital Universitario 12 de Octubre, Madrid, Spain; g Department of Medical Oncology, Hospital Clinic, Barcelona, Spain; Translational Genomics and Targeted Therapies in Solid Tumors, IDIBAPS, Barcelona, Spain; Department of Medicine, University of Barcelona, Barcelona, Spain; h UGCI Oncología Médica Hospitales Regional y Virgen de la Victoria, Málaga, Spain; i Hospital Universitario Virgen de la Macarena, Sevilla, Spain; j Hospital Universitario Ramón y Cajal, Madrid, Spain; k Institut Català d’Oncologia, Hospital Duran i Reynals, Barcelona, Spain; l Hospital Universitario de Jerez, Jerez, Spain; m Hospital Universitario Miguel Servet, Zaragoza, Spain; n Hospital Universitario Donostia-BioDonostia, Donostia, Spain; o Fundación Instituto Valenciano de Oncología, Valencia, Spain; p Departamento de Oncología, Badalona-Applied Research Group in Oncology (B-ARGO Group), Institut Català d’Oncologia, Badalona, Spain; q Hospital Universitario Insular Gran Canaria, Gran Canaria, Spain; r Instituto Maimonides de Investigacion Biomedica (IMIBIC), Hospital Reina Sofía, Universidad de Córdoba, Spain; s Hospital Universitario San Pedro de Alcántara, Cáceres, Spain; t Hospital Universitario San Juan de Alicante, Alicante, Spain; u Hospital Universitari Santa Creu i Sant Pau and CIBERONC Breast Cancer Programme, Department of Medicine, Universitat Autonoma Barcelona, Barcelona, Spain; v Hospital Puerta de Hierro Majadahonda, Madrid, Spain; w Hospital Universitario Infanta Cristina, Badajoz, Spain; x Hospital Universitari Vall d’Hebron, Barcelona and Institut Oncològic Vall d’Hebron (VHIO), Barcelona, Spain; y Novartis Pharmaceuticals Spain, Barcelona, Spain; z Hospital Universitario Gregorio Marañón, Madrid, Spain

**Keywords:** Advanced breast cancer, Ribociclib, CDK4/6 inhibitor, Postmenopausal, Premenopausal, ABC, advanced breast cancer, AE, adverse event, AESI, adverse event of special interest, ALT, alanine aminotransferase, AST, aspartate aminotransferase, CBR, clinical benefit rate, CDK4/6, cyclin-dependent kinase 4/6, CI, confidence interval, CNS, central nervous system, CR, complete response, ECOG, Eastern Cooperative Oncology Group, ET, endocrine therapy, FACT-B, Functional Assessment of Cancer Therapy–Breast Cancer, FAS, full analysis set, FPFV, First Patient First Visit, HER2−, human epidermal growth factor receptor 2-negative, HR+, hormone receptor-positive, HRQoL, health-related quality of life, LPFV, last patient first visit, LPLV, last patient last visit, NE, not estimable, ORR, overall response rate, OS, overall survival, PFS, progression-free survival, PR, partial response, PRO, patient-reported outcome, QTcF, QT interval corrected for heart rate using Fridericia's formula, RECIST, Response Evaluation Criteria in Solid Tumors, SAE, serious adverse event, SD, stable disease, SS, safety set, TTP, time to progression

## Abstract

**Background:**

Breast cancer is the most common malignancy and the second leading cause of cancer-related mortality in Spanish women. Ribociclib in combination with endocrine therapy (ET) has shown superiority in prolonging survival in patients with hormone receptor-positive (HR+), human epidermal growth factor receptor 2-negative (HER2−) advanced breast cancer (ABC) vs. ET alone.

**Methods:**

CompLEEment-1 is a single-arm, open-label phase 3b trial evaluating ribociclib plus letrozole in a broad population of patients with HR+, HER2– ABC. The primary endpoints were safety and tolerability. Here we report data for Spanish patients enrolled in CompLEEment-1.

**Results:**

A total of 526 patients were evaluated (median follow-up: 26.97 months). Baseline characteristics showed a diverse population with a median age of 54 years. At study entry, 56.5% of patients had visceral metastases and 8.7% had received prior chemotherapy for advanced disease. Rates of all-grade and Grade ≥3 adverse events (AEs) were 99.0% and 76.2%, respectively; 21.3% of patients experienced a serious AE, and 15.8% of AEs led to treatment discontinuation. AEs of special interest of neutropenia, increased alanine aminotransferase, increased aspartate aminotransferase and QTcF prolongation occurred in 77.8%, 14.8%, 11.4% and 4.0% of patients, respectively. Patients aged >70 years experienced increased rates of all-grade and Grade ≥3 neutropenia and anemia. Efficacy results were consistent with the global study.

**Conclusions:**

Results from Spanish patients enrolled in CompLEEment-1 are consistent with global data showing efficacy and a manageable safety profile for ribociclib plus letrozole treatment in patients with HR+, HER2− ABC, including populations of interest (NCT02941926).

**Trial registration:**

ClinicalTrials.gov NCT02941926

## Highlights

• Results from the CompLEEment-1 Spanish population are comparable to the global results.
• Manageable safety was shown in elderly patients and those with visceral disease.
• Efficacy data were comparable to those of the global study in a younger population.
• Efficacy and safety of ribociclib are confirmed in a broader patient population.
• Results confirm a robust response to treatment in a local setting.

## 1 Introduction

Breast cancer is the most common malignancy in Spanish women, with approximately 33,000 new cases estimated to be diagnosed in 2021; it is also the second leading cause of cancer-related mortality for Spanish women [1]. Invasive breast cancer cases represent just under 30% of all invasive cancers diagnosed in Spanish women [2,3]. Breast cancer rates in Spain have remained stable over the past decades with an incidence of ∼88 per 100,000, which is lower than the European average (108.8 per 100,000) [2]. Approximately 19% of women diagnosed with breast cancer in Spain were aged under 45 years [4]. Although tumor biology in younger, premenopausal women tends to be more aggressive than in older ones, these women are often underrepresented in clinical trials [5,6].

Hormone receptor-positive (HR+) breast cancer is the most common subtype, representing up to 75% of breast cancer cases [7,8]. Endocrine therapy (ET) is a long-established first-line treatment for HR + advanced breast cancer (ABC); however, the effectiveness of ET is limited by the development of endocrine resistance, which prevents patients from achieving long-term clinical benefit [9]. The combination of ET with cyclin-dependent kinase 4/6 (CDK4/6) inhibitors has resulted in prolonged clinical benefit in patients with HR+, human epidermal growth factor receptor 2-negative (HER2−) ABC, and is now the recommended first-line treatment for these patients as per the ESO-ESMO and NCCN guidelines [10,11].

Ribociclib, an orally bioavailable, highly selective CDK4/6 inhibitor, is currently approved by Spanish Health Authorities in combination with an aromatase inhibitor or fulvestrant as first-line treatment for pre-/postmenopausal patients with HR+, HER2− ABC [12]. The phase 3 MONALEESA-2, -3 and -7 trials have established the efficacy and safety of ribociclib in combination with ET in patients with HR+, HER2− ABC [[13], [14], [15], [16], [17]], showing superiority vs. ET alone in prolonging both progression-free survival (PFS) and overall survival (OS), regardless of ET partner, line of therapy, or menopausal status.

Despite these positive results, evidence for the safety and efficacy of ribociclib plus ET in a broader patient population is lacking, as patients with poor performance status, central nervous system (CNS) metastases and those who have received chemotherapy for ABC are frequently excluded from clinical trials. The phase 3b CompLEEment-1 trial assessed the safety and tolerability of ribociclib plus letrozole in a larger and broader population of patients with HR+, HER2− ABC than those eligible for previous phase 3 trials [18]. Unlike MONALEESA-2 and MONALEESA-7, the CompLEEment-1 trial population included patients who were male, had CNS metastases, and/or an Eastern Cooperative Oncology Group (ECOG) performance status of 2. Furthermore, CompLEEment-1 also included patients who had received prior chemotherapy for advanced disease, who are frequently excluded from clinical trials despite clinical data showing that a significant number of patients with HR+, HER2– ABC receive chemotherapy as first-line treatment [19]. Safety and efficacy data for ribociclib in CompLEEment-1 were consistent with those of MONALEESA-2 and -7; the median time to progression was 27.1 months (95% confidence interval [CI], 25.7 to not reached) [18].

Here, we present a detailed analysis of the efficacy and safety of ribociclib plus letrozole in a broad population of Spanish patients with HR+, HER2− ABC enrolled in the CompLEEment-1 trial.

## 2 Methods

### 2.1 Study design and treatment

CompLEEment-1 (NCT02941926) is an open-label, single arm, multicenter phase 3b study assessing the overall safety, tolerability, and clinical efficacy of ribociclib in combination with letrozole in men, and pre- and postmenopausal women with HR+, HER2− ABC who did not receive prior ET for advanced disease [18]. The study consists of 2 phases (Supplementary Fig. 1): a core phase running from the First Patient First Visit (FPFV) until 18 months after the Last Patient First Visit (LPFV), and an extension phase running from 18 months after LPFV (end of the core phase) to the Last Patient Last Visit (LPLV). Patients transitioned to the extension phase only if they were still obtaining clinical benefit from the treatment at the end of the core phase and had no access to ribociclib outside of the clinical trial.

Patients received ribociclib at a total daily starting dose of 600 mg (3 tablets of 200 mg) on a 3 weeks on/1 week off schedule, with or without food. Additionally, patients received 2.5 mg letrozole orally once a day on a continuous schedule through a 28-day cycle. Male patients and premenopausal female patients also received 3.6 mg goserelin (as an injectable subcutaneous implant) or 7.5 mg leuprolide (as an intramuscular injection) once per cycle. Treatment continued until disease progression, unacceptable toxicity, death, or discontinuation from study treatment for any reason. All patients were followed for 30 days following the last ribociclib dose. Dose reduction, dose interruption, and/or discontinuation of ribociclib were permitted for the management of severe adverse events (AEs), whereas dose reductions were not permitted for letrozole, goserelin or leuprolide [18]. Data cutoff was 8 November 2019.

### 2.2 Key inclusion and exclusion criteria

Eligible patients included adult males, pre- and postmenopausal females aged ≥18 years with locoregionally recurrent or metastatic HR+, HER2– ABC not amenable to curative therapy. Only those patients who had received ≤1 line of chemotherapy and no prior ET for advanced disease were enrolled in this study. Patients with a treatment-free interval (TFI) > 12 months from completion of (neo)adjuvant therapy with letrozole or anastrozole and those who had received treatment in the metastatic setting with letrozole or anastrozole for ≤28 days prior to enrollment were eligible. All enrolled patients displayed adequate bone marrow and organ function and had an ECOG performance status of ≤2. At screening, eligible patients had corrected QT interval (QTc) <450 ms as measured by Fridericia's correction (QT interval corrected for heart rate using Fridericia's formula, QTcF), and a resting heart rate of ≥50 beats per minute.

Key exclusion criteria included receipt of any prior CDK4/6 inhibitor or systemic hormonal therapy for ABC or concurrent use of other anticancer therapy; known history of HIV infection; clinically significant, uncontrolled heart disease and/or cardiac repolarization abnormalities; and concurrent malignancy or malignancy within 3 years prior to starting study drug (except requisitely treated basal cell or squamous cell carcinoma, non-melanoma skin cancer; or curatively resected cervical cancer). Patients with CNS metastases were also excluded, unless the patient had completed any prior therapy for CNS disease 4 weeks before the start of study treatment and CNS lesions were clinically stable.

### 2.3 Endpoints and study assessments

The primary objective of this study was to assess the safety and tolerability of ribociclib in combination with letrozole in a broad patient population. Primary endpoints included the number of patients experiencing any adverse events (AEs); grade 3/4 AEs; serious AEs (SAEs); adverse events of special interest (AESIs); AEs leading to dose reduction, interruption, or discontinuation; and AE-related deaths. AESIs were defined according to ongoing reviews of ribociclib safety data, and included neutropenia, QTcF prolongation, and hepatobiliary toxicity (measured as elevation of aspartate aminotransferase [AST] and alanine aminotransferase [ALT] blood levels). Safety assessments involved monitoring and recording all AEs, grade 3/4 AEs and SAEs, AESIs, AEs leading to drug discontinuation and deaths. MedDRA version 22.1 and CTCAE version 4.03 were used to define AESIs and AEs (preferred term), respectively.

Secondary endpoints related to the clinical efficacy of ribociclib plus letrozole included time-to-progression (TTP) based on investigators’ assessment (defined as time from date of treatment initiation to the date of event); overall response rate (ORR) for patients with measurable disease (defined as the proportion of patients with a best overall response of complete response [CR] or partial response [PR]); and clinical benefit rate (CBR), defined as the proportion of patients with a best overall response of CR or PR, or an overall lesion response of stable disease (SD), lasting for at least 24 weeks, as per local review. Tumor response, which was assessed locally, was based on Response Evaluation Criteria in Solid Tumors (RECIST) v1.1 criteria. Tumor assessments were performed according to the current standard of care; assessments were recommended to take place every 12 weeks until disease progression.

Another secondary endpoint was health-related quality of life (HRQoL) using the Functional Assessment of Cancer Therapy–Breast Cancer (FACT-B) questionnaire [20], translated into Spanish. This was only completed by female patients due to the nature of the questionnaire. Responses to the FACT-B questionnaire were collected using electronic devices at Day 1 of each cycle up to Cycle 6, every 2 cycles up to Cycle 12 and every 3 cycles thereafter.

### 2.4 Statistical analysis

Demographic and other baseline data including disease characteristics were summarized descriptively for the Full Analysis Set (FAS) of the Spanish patients’ subgroup. The FAS included all patients who received at least one dose of study treatment (either ribociclib or letrozole or goserelin/leuprolide [if applicable] in the core phase).

The safety analysis was conducted using the Safety Set (SS) of the Spanish patients’ subgroup, which included all patients in the FAS. The primary safety variables (AEs, SAEs, AESIs, AEs leading to dose reduction or interruption, and AEs leading to discontinuation and deaths) were summarized by count and percentage.

The clinical efficacy analysis was conducted using the FAS. ORR and CBR were calculated and summarized using frequency tables with associated 2-sided exact 95% CI, whereas TTP was estimated using the Kaplan-Meier method. The patient-reported outcome (PRO) analysis set consisted of all female patients in the FAS population for whom baseline and at least one postbaseline measurements were available.

### 2.5 Ethics

The study was designed, implemented, and reported in accordance with the International Council for Harmonisation of Technical Requirements for Pharmaceuticals for Human Use (ICH) Harmonized Tripartite Guidelines for Good Clinical Practice, with applicable local regulations, and with the ethical principles laid down in the Declaration of Helsinki. The protocol and informed consent form were reviewed and approved by a properly constituted Institutional Review Board/Independent Ethics Committee/Research Ethics Board before study commencement. Written informed consent was obtained from all patients. A steering committee oversaw the conduct of the trial as per the approved protocol. Representatives of the trial sponsor, Novartis Pharmaceuticals (East Hanover, NJ), collected and analyzed the data.

## 3 Results

### 3.1 Patient demographics and baseline characteristics

Patient disposition and baseline characteristics of all patients enrolled in the global study have been described previously [18]. Overall, 526 Spanish patients were enrolled and received at least one dose of study treatment between April 2017 and November 2019. The median follow-up time was 26.97 months (range, 21.4–33.84). The median duration of exposure to ribociclib was 18.6 months, while this was 18.8 months for letrozole (n = 526) and 16.8 months for goserelin (n = 173); no Spanish patients received leuprolide. The median average daily dose of ribociclib was 600.0 mg (240.0–600.0), while the median dose intensity was 576.7 mg/day (204.5–682.4). Overall, 58.0% of patients discontinued study treatment (Supplementary Table 1); the most common reasons for treatment discontinuation were progressive disease (33.8%) and AEs (15.0%).

Baseline patient characteristics showed a diverse population in terms of age and disease characteristics (Table 1, Table 2). The median age was 54 years (range, 24–85), and 15.8% of patients were aged >70 years (Table 1). The vast majority of patients were female (99.2%, with 4 male patients [0.8%] enrolled), and 64.4% were postmenopausal women; most patients (97.7%) had an ECOG performance status of ≤1.

**Table 1:** Baseline patient characteristics.

Characteristic	All Patients N = 3246	Spanish Patients N = 526
**Median age, years** (range)	58.0 (20–92)	54.0 (24–85)
**Age category, years**, n (%)		
<70 years	2613 (80.5)	443 (84.2)
70 to ≥75 years	633 (19.5)	83 (15.8)
**Gender**, n (%)		
Female	3207 (98.8)	522 (99.2)
Female, postmenopausal	2485 (76.6)	339 (64.4)
**ECOG PS**, n (%)		
0	1964 (60.5)	346 (65.8)
1	1161 (35.8)	168 (31.9)
2	112 (3.5)	7 (1.3)
Missing	9 (0.3)	5 (1.0)

ECOG PS, Eastern Cooperative Oncology Group Performance Status.

**Table 2:** Disease characteristics.

Characteristic	All Patients N = 3246	Spanish Patients N = 526
**Median time since initial diagnosis**, months (range)	42.5 (0.1–469.9)	46.8 (0.1–412.8)
**Disease-free interval**, n (%)		
*De novo*^a^	1041 (32.1)	158 (30.0)
Non-*de novo*^b^	2201 (67.8)	368 (70.0)
≤24 months	382 (11.8)	62 (11.8)
>24 months	1819 (56.0)	306 (58.2)
**Hormone receptor status**, n (%)		
Estrogen receptor-positive	3231 (99.5)	525 (99.8)
Progesterone receptor-positive	2608 (80.3)	417 (79.3)
**Site of metastases**, n (%)		
Bone	2409 (74.2)	401 (76.2)
Bone-only	704 (21.7)	151 (28.7)
Breast	183 (5.6)	29 (5.5)
CNS	51 (1.6)	8 (1.5)
Visceral	1992 (61.4)	297 (56.5)
Liver	862 (26.6)	143 (27.2)
Lung	1416 (43.6)	196 (37.3)
Other	295 (9.1)	36 (6.8)
Skin	110 (3.4)	8 (1.5)
Lymph nodes	1250 (38.5)	180 (34.2)
Other	163 (5.0)	15 (2.9)
**Metastatic sites**, n (%)		
0	15 (0.5)	2 (0.4)
1	903 (27.8)	175 (33.3)
2	923 (28.4)	153 (29.1)
3	644 (19.8)	99 (18.8)
4	375 (11.6)	55 (10.5)
≥5	386 (11.9)	42 (8.0)
**Prior (neo)adjuvant ET**, n (%)		
Anti-estrogen^c^	1156 (35.6)	207 (39.4)
Aromatase inhibitors^d^	1091 (33.6)	117 (22.2)
**Prior chemotherapy for advanced disease**, n (%)	194 (6.0)	46 (8.7)

a *De novo* includes patients with no date of first recurrence/progression or with a first recurrence/progression within 90 days of initial diagnosis without prior antineoplastic medication.

b Non-*de novo* disease was calculated as the time from initial diagnosis to first recurrence/progression, categorized as ≤12 months, >12 to ≤24 months, and ≥24 months.

c Includes tamoxifen.

d Includes anastrozole, exemestane and letrozole. CNS, central nervous system; ET, endocrine therapy.

At baseline, 56.5% of patients had visceral metastases, whereas 1.5% of patients had CNS metastases (Table 2). Overall, 37.3% of patients had ≥3 metastatic sites at baseline, whereas 8.7% of patients had received prior chemotherapy for advanced disease.

### 3.2 Safety

Nearly all patients experienced an AE (99.0%), with Grade ≥3 AEs occurring in 76.2% of patients (Table 3) and all-grade SAEs occurring in 21.3% (Grade ≥3 SAEs, 17.7%). Only 15.8% of all-grade AEs (Grade ≥3, 8.9%) led to treatment discontinuation, while 74.1% (Grade ≥3, 66.5%) required dose adjustment or interruption.

**Table 3:** Safety overview.

Category	Spanish Patients, N = 526	
	All Grades n (%)	Grade ≥3 n (%)
AEs	521 (99.0)	401 (76.2)
Treatment-related	494 (93.9)	352 (66.9)
SAEs	112 (21.3)	93 (17.7)
Treatment-related	19 (3.6)	16 (3.0)
Fatal SAEs	10 (1.9)	10 (1.9)
Treatment-related	2 (0.4)	2 (0.4)
AEs leading to discontinuation	83 (15.8)	47 (8.9)
Treatment-related	63 (12.0)	36 (6.8)
AEs leading to dose adjustment/interruption	390 (74.1)	350 (66.5)
Treatment-related	351 (66.7)	324 (61.6)

A patient with multiple severity grades for an AE is only counted under the maximum grade. AE, adverse event; SAE, serious adverse event.

The majority of AEs were assessed as treatment-related (all grade, 93.9%; Grade ≥3, 66.9%), but the rates of SAEs assessed as related to treatment were low (all grade, 3.6%; Grade ≥3, 3.0%). Two deaths were assessed as resulting from treatment-related AEs: the causes were pneumonitis, and pancytopenia and sepsis (it should be noted that the latter patient was concomitantly receiving metamizole).

The most common AEs reported were neutropenia (all grade, 77.4%; Grade ≥3, 59.5%), asthenia (all grade, 37.8%; Grade ≥3, 1.9%), nausea (all grade, 28.1%; Grade ≥3, 0.6%), arthralgia (all grade, 24.0%; Grade ≥3, 0%), and anemia (all grade, 20.3%; Grade ≥3, 1.9%) (Table 4). Rates of all-grade neutropenia were similar across patient subgroups of interest (patients aged >70 years, patients who received prior chemotherapy for advanced disease, and patients with visceral metastases at diagnosis), although increased proportions of patients aged >70 years experienced Grade ≥3 neutropenia (64.0% vs. 59.5% in all patients) (Table 4). The reported frequency of anemia was also increased in patients aged >70 years compared with all patients (all grade, 37.3% vs. 20.3%; Grade ≥3, 5.3% vs. 1.9%). Increased proportions of these elderly patients also experienced constipation (22.7% vs. 16.9%), alopecia (26.7% vs. 16.7%), vomiting (22.7% vs. 15.4%), and decreased appetite (25.3% vs. 11.0%) compared with the whole patient population; the majority of these events were grade 1 or 2. Meanwhile, patients who had received prior chemotherapy for advanced disease reported increased rates of all-grade arthralgia (32.6% vs. 24.0% in all patients).

**Table 4:** Adverse events reported in >15% of patients in subgroups of interest.

Preferred Term	All patients N = 526		Age >70 years N = 75		Prior chemotherapy N = 46		Visceral metastases N = 297	
	All Grades n (%)	Grade ≥3 n (%)	All Grades n (%)	Grade ≥3 n (%)	All Grades n (%)	Grade ≥3 n (%)	All Grades n (%)	Grade ≥3 n (%)
Number of patients with >1 event	521 (99.0)	401 (76.2)	75 (100.0)	63 (84.0)	45 (97.8)	31 (67.4)	293 (98.7)	224 (75.4)
Neutropenia	407 (77.4)	313 (59.5)	58 (77.3)	48 (64.0)	35 (76.1)	24 (52.2)	226 (76.1)	169 (56.9)
Asthenia	199 (37.8)	10 (1.9)	31 (41.3)	4 (5.3)	10 (21.7)	0	109 (36.7)	7 (2.4)
Nausea	148 (28.1)	3 (0.6)	21 (28.0)	0	12 (26.1)	0	89 (30.0)	2 (0.7)
Arthralgia	126 (24.0)	0	12 (16.0)	0	15 (32.6)	0	67 (22.6)	0
Anemia	107 (20.3)	10 (1.9)	28 (37.3)	4 (5.3)	9 (19.6)	1 (2.2)	63 (21.2)	8 (2.7)
Constipation	89 (16.9)	1 (0.2)	17 (22.7)	1 (1.3)	4 (8.7)	0	47 (15.8)	1 (0.3)
Alopecia	88 (16.7)	0	20 (26.7)	0	1 (2.2)	0	48 (16.2)	0
Diarrhea	83 (15.8)	4 (0.8)	12 (16.0)	0	9 (19.6)	1 (2.2)	45 (15.2)	2 (0.7)
Vomiting	81 (15.4)	4 (0.8)	17 (22.7)	1 (1.3)	6 (13.0)	0	46 (15.5)	2 (0.7)
Decreased appetite	58 (11.0)	1 (0.2)	19 (25.3)	1 (1.3)	1 (2.2)	0	36 (12.1)	1 (0.3)

A patient with multiple severity grades for an adverse event is only counted under the maximum grade.

Most cases of neutropenia were managed with dose interruption or dose reduction, while nearly half of hypertransaminasemia cases were managed with dose reduction; on the other hand, few cases of QTcF prolongation required this approach (Table 5). The numbers of patients who required permanent drug withdrawal due to neutropenia, ALT increase, AST increase and QTcF prolongation were 4 (0.8%), 29 (5.5%), 25 (4.8%) and 0, respectively. The rates of recovery/resolution were greater than the rates of non-recovery/non-resolution for all AESIs except transaminase increase. AESIs very rarely led to hospitalization (0–0.2% of patients) and no fatal AESIs were recorded.

**Table 5:** Adverse events of special interest.

n (%)^a^	Neutropenia	Liver Enzyme Elevation		QTcF Prolongation^b^
		ALT	AST	
All-grade events	409 (77.8)	78 (14.8)	60 (11.4)	21 (4.0)
Leading to dose interruption	299 (56.8)	34 (6.5)	25 (4.8)	2 (0.4)
Leading to dose reduction	90 (17.1)	6 (1.1)	3 (0.6)	2 (0.4)
Leading to dose withdrawal	4 (0.8)	29 (5.5)	25 (4.8)	0
Leading to hospitalization	0^c^	0	0	1 (0.2)
Medication or therapy taken	15 (2.9)	2 (0.4)	4 (0.8)	1 (0.2)
Not recovered/not resolved	242 (46.0)	44 (8.4)	35 (6.7)	2 (0.4)
Recovering/resolving	223 (42.4)	37 (7.0)	34 (6.5)	1 (0.2)
Recovered/resolved	376 (71.5)	46 (8.7)	33 (6.3)	19 (3.6)
With sequelae	9 (1.7)	0	1 (0.2)	0
Leading to death	0	0	0	0

a Percentage value calculated based on 526 patients. A patient is counted no more than once in each AE outcome. If a patient has AEs with different outcomes, the patient will be counted in several outcomes. If the patient has several events with the same outcome, he/she will be counted only once in the corresponding outcome line.

b Includes “Electrocardiogram QT prolonged”, “Electrocardiogram QT interval abnormal”, “Syncope” and “Long QT syndrome”.

c 4 (0.8) cases with febrile neutropenia led to hospitalization. AE, adverse event; AESI, adverse event of special interest; ALT, alanine aminotransferase; AST, aspartate aminotransferase; QTcF, QT interval corrected for heart rate using Fridericia's formula.

At data cutoff, 10 patients (1.9%) had died, most of them as a result of disease progression (n = 5, 1.0%). Other causes of death were embolism, hepatic failure, pneumonitis, respiratory failure and sepsis (one patient each); of these, the deaths due to pneumonitis and sepsis were assessed as related to treatment.

### 3.3 Efficacy

**Fig. 1:**
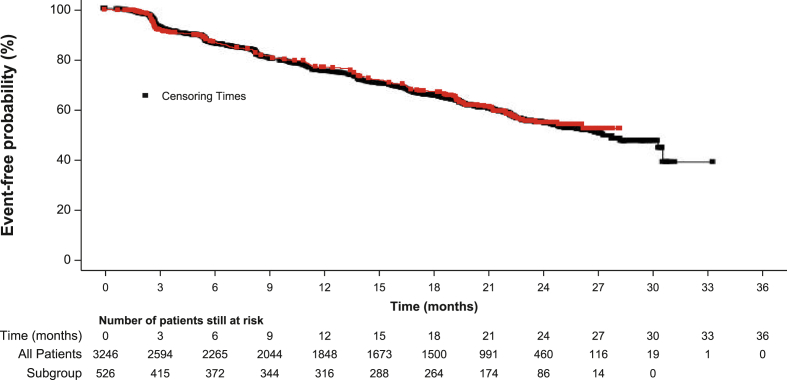
Time to progression in Spanish patients and all patients enrolled in the CompLEEment-1 study. Red, Spanish patients; black, all patients enrolled in the global study. Squares denote censored events. CI, confidence interval; NE, not estimable.

The median TTP was not estimable (NE; 95% CI: 23.5, NE; n = 180), while the Kaplan-Meier estimated event-free probability was 77.0% (95% CI: 72.8, 80.7) at 12 months and 54.9% (95% CI: 49.5, 60.0) at 24 months (Fig. 1).

ORR and CBR for patients with measurable disease at baseline were 46.6% (95% CI: 40.9, 52.5) and 68.5% (95% CI: 62.8, 73.7), respectively (Supplementary Table 2). Overall, 3.4% of patients experienced a CR and 43.3% a PR. ORR and CBR were higher for patients aged <50 years than for those aged ≥50 years. Patients aged <50 years also had increased CR rates (5.1% vs. 2.5% for age ≥50 years). In terms of menopausal status, ORR and CBR were similar for pre- and postmenopausal women, although CR rates were higher for premenopausal women (Supplementary Table 2). Metastatic status also affected efficacy rates, with patients presenting with visceral metastases having higher ORR rates compared with patients with no visceral metastases at baseline (47.7% vs 43.6%, respectively; Supplementary Table 3); the number of metastatic sites also affected efficacy rates, with patients with <3 metastatic sites more likely to experience a CR (5.2% vs. 1.4% for patients with ≥3 metastatic sites at baseline). Finally, patients with no prior chemotherapy treatment for advanced disease had substantially higher ORR and CBR rates compared with those exposed to chemotherapy at baseline (48.5% and 69.5% vs 31.3% and 59.4%, respectively), largely driven by higher rates of PR (45.1% vs. 28.1% in patients with prior chemotherapy treatment).

In terms of PROs, HRQoL remained stable throughout the study without any major changes (Supplementary Fig. 2); median (SD) FACT-B scores were recorded as 101.2 (16.45) at baseline and 93.3 (19.65) at end of treatment, with a median change from baseline of −7.3.

## 4 Discussion

Our results support the clinical safety, tolerability and efficacy of treatment with ribociclib in combination with letrozole in a broad population of Spanish patients with HR+, HER2− ABC who had not previously received ET for their advanced disease. Overall, the data are consistent with those reported in the global CompLEEment-1 study [18] and support the use of ribociclib plus letrozole in the first-line setting for patients with HR+, HER2− ABC. Moreover, these results demonstrate the reproducibility and robustness of treatment with ribociclib and letrozole in this patient population across different environments and local settings.

The Spanish patient population enrolled in CompLEEment-1 represented 14% of the global population of the study and reflected a broad group of patients with HR+, HER2− ABC that was more representative of a real-world patient population than subjects included in other randomized phase 3 trials of CDK4/6 inhibitors in combination with aromatase inhibitors; in particular, a substantial proportion of patients in this trial had received chemotherapy for advanced disease [13,15,21]. Interim safety results from the Spanish population subset in CompLEEment-1 reported using an earlier cut-off date were consistent with previous data from MONALEESA-2, MONALEESA-7, and the CompLEEment-1 global cohort, and confirmed the predictable and manageable safety profile of ribociclib in combination with letrozole as first-line treatment for patients with HR+, HER2− ABC [22]. Spanish CompLEEment-1 patients had a lower median age compared with those enrolled in the global study (54.0 vs 58.0 years, respectively); the proportion of patients aged <65 years was also larger among the Spanish subset (76.0% vs 66.9% in the global study). Consistent with this observation, a lower proportion of Spanish patients were postmenopausal (64.4% vs 76.6% in the global study). Similar proportions of patients had ECOG ≤1 (97.7% vs 96.3% in the global study), while more Spanish patients had <3 metastatic sites at baseline (62.7% vs 56.7% in the global study). Overall, 8.7% of Spanish patients had received chemotherapy for advanced disease, compared with 6.0% in the global study; this slightly higher percentage could reflect a more aggressive treatment approach for Spanish women, which could be explained by the Spanish population being younger on average than the overall study population.

The reported AE profile among Spanish CompLEEment-1 patients was consistent with that reported in previous ribociclib trials [13,15], and no new safety signals were identified. Rates of AEs and SAEs were similar to those reported for the global study (99.0% and 21.3% vs 98.7% and 21.6%, respectively), as were the rates of treatment discontinuation due to AEs (15.8% vs 16.3% in the global study). This was despite a numerically, albeit not significantly higher median dose intensity (576.7 mg/day [204.5–682.4] vs 571.4 mg/day [120.0–800.0] in the global study).

Neutropenia was the most common AE reported in Spanish patients, as in the global study, followed by asthenia and nausea. Spanish patients aged >70 years reported increased rates of all-grade and grade ≥3 neutropenia and anemia, as well as increased rates of certain non-hematologic AEs (constipation, alopecia, vomiting and decreased appetite). These results are similar to those from patients aged ≥65 years enrolled in the MONALEESA-2 trial [23]; these patients experienced increased rates of neutropenia and anemia, and the most frequent non-hematologic AEs (including nausea, fatigue, alopecia, vomiting, and diarrhea) were predominantly grade 1–2. Our results support the safety of ribociclib in combination with letrozole in Spanish patients aged >70 years, a population that is under-represented in clinical trials and which may present challenges to treatment due to a high incidence of pre-existing comorbidities [24,25]. No notable differences in rates of the most common AEs were detected for patients who had received prior chemotherapy for advanced disease or those with visceral metastases at baseline, supporting the safety of ribociclib plus letrozole in these patient populations.

AESIs were managed by dose reduction or interruption, and rarely led to hospitalization. The rates of resolved events were higher than rates of non-resolved events for all AESIs except ALT and AST increase. The relatively high percentage of non-resolved or resolving AESIs could be due to the mild nature of the AEs; in the case of ALT/AST increase, this could also be explained by the resolution time determined in the protocol (28 days) being too short to allow for complete resolution of transaminitis.

Median TTP was not estimable, but the Kaplan-Meier estimated probabilities at 12 and 24 months among Spanish CompLEEment-1 patients were similar to those reported in the global study (77.0% at 12 months and 54.9% at 24 months vs 75.1% at 12 months and 54.7% at 24 months, respectively). The lack of event-free survival data at 33 months is likely due to the fact that the study opened in Spain later than in other locations. Median PFS was 26.7 months (95% CI: 24.8, 30.1) for the total population in the global CompLEEment-1 study, with a median follow-up of 25.4 months [18]; this was comparable to the median PFS reported for MONALEESA-2 (25.3 months [95% CI: 23.0, 30.3] with a median follow-up of 26.4 months) and MONALEESA-7 (23.8 months [95% CI: 19.2, not reached], with a median follow-up of 19.2 months). The similarity of median PFS measures across studies is of note as the CompLEEment-1 study enrolled a much broader patient population, including patients who had received chemotherapy for advanced disease [13,15]; these results highlight the efficacy of ribociclib in a patient population more representative of real-world patients.

ORR and CBR for Spanish patients with measurable disease at baseline were similar to those reported in the global study (46.6% and 68.5% vs 43.6% and 69.1%, respectively). The ORR was lower than those reported in the MONALEESA-2 and MONALEESA-7 trials for patients with measurable disease at baseline (54.5% and 51.0%, respectively) [13,15]. This may be due to the differences in patient population enrolled in CompLEEment-1, although it should also be noted that response assessment in this study was not centralized, but rather depended on the local standard of care. Age <50 years, presence of visceral metastases at baseline, <3 metastatic sites and no prior chemotherapy treatment for advanced disease all correlated with higher ORR and CBR in Spanish patients, while rates were similar for pre- and postmenopausal women. It should be noted that patients with visceral metastases are more likely to have measurable disease and thus be evaluable for response, which could explain the higher ORR and CBR rates in patients with visceral metastases at baseline. FACT-B scores were maintained relative to baseline, reflecting preservation of HRQoL.

A potential limitation of CompLEEment-1 is that tumor assessments were performed according to the current standard of care in different locations; response assessment timings may have varied, with different intervals according to the local standard of care. However, we believe this is an actual strength of the study, showing the robustness of response to ribociclib and supporting the utility of this type of analysis in a local population. Efficacy and PRO data should be interpreted with some caution given the lack of randomization and a control arm.

Overall, these findings support the efficacy and manageable safety profile of ribociclib in combination with letrozole as first-line treatment in a population of Spanish patients with HR+/HER2− ABC approaching that of a real-world setting.

## Funding

This study was funded by Novartis Pharmaceuticals. The sponsor was involved in the study design, the collection, analysis, and interpretation of data, and the writing of the manuscript.

## Conflict of interest/disclosures

JSB has received research grants from AstraZeneca, Lilly, Pfizer and Roche; consulting fees from AstraZeneca, Daiichi Sankyo, Gilead, Lilly, Pfizer, Roche and Seagen; honoraria from Lilly, Novartis and Pfizer. FMA has received research grants from Pfizer; consulting fees from AstraZeneca, Daiichi Sankyo, Eisai, MSD, Pfizer, Roche and Seagen; honoraria from Pfizer; travel grants from Daiichi Sankyo, Novartis and Pfizer. EGC has received research grants from Pfizer; consulting fees from AstraZeneca, Daiichi Sankyo, Gilead, Pfizer, Pharmamar, Roche and Seagen; honoraria from Eisai, Novartis and Pfizer; travel grants from Novartis, Pfizer and Roche. CHM has received honoraria from Novartis; travel grants from Novartis, Pfizer and Roche. EMCG has received honoraria from AstraZeneca, Daiichi Sankyo, Lilly, Novartis, Pfizer, Roche and Seagen; travel grants from AstraZeneca, Daiichi Sankyo, Novartis and Pfizer; has participated in advisory boards for AstraZeneca, Daiichi Sankyo, Lilly, Novartis, Pfizer, Roche and Seagen. MV has received honoraria from Daiichi Sankyo, Novartis, Pfizer and Roche; travel grants from Pfizer and Roche; has participated in advisory boards for Novartis and Roche. LDLCM has received honoraria from AstraZeneca, BMS, Incyte, MSD and Roche; travel grants from Roche. RVV has received consulting fees from Novartis; honoraria from Eisai, Lilly and Novartis; payment for expert testimony from Novartis; travel grants from Lilly, Novartis and Roche; has participated in advisory boards for Novartis. AAT has taken advisory/consultancy roles for Eli Lilly and Gilead; has received honoraria from AstraZeneca-Daichii-Sanykio, Eli Lilly, Pfizer and Seagen. IMAL has received institutional grants from Novartis; honoraria from Novartis. JGG has received honoraria from AstraZeneca, Daiichi Sankyo, Lilly, Novartis, Pfizer, Roche and Seagen; travel grants from AstraZeneca, Daiichi Sankyo, Lilly, Novartis, Pfizer, Roche and Seagen; has participated in advisory boards for AstraZeneca, Daiichi Sankyo, Lilly, Novartis, Pfizer, Roche and Seagen. VQG has been an invited speaker for Novartis and is a steering committee member for Roche. EVR has received honoraria from Lilly, Novartis, Pfizer and Roche; travel grants from Lilly, Novartis, Pfizer and Roche. JDLHR has received consulting fees from Lilly, Novartis, Pfizer, Roche and Seagen; honoraria from Lilly, Novartis, Pfizer, Roche and Seagen; payment for expert testimony from Lilly, Novartis, Pfizer, Roche and Seagen; travel grants from Lilly, Novartis, Pfizer, Roche and Seagen; has patents planned, issued or pending with Roche; has participated in advisory boards for Lilly, Novartis, Pfizer, Roche and Seagen. SGS has received honoraria from AstraZeneca, Clovis, GSK, Novartis and Pfizer; travel grants from GSK, Pfizer and Roche; has participated in advisory boards for AstraZeneca, GSK, Lilly and Seagen. ABM has received institutional grants from Bristol Myers Squibb, Lilly, MSD, Novartis, Pfizer and Roche; consulting fees from AstraZeneca, Exact Sciences, Gilead, Lilly, MSD, Pfizer, Roche and Seagen; honoraria from AstraZeneca, Novartis, Pfizer and Roche; travel grants from Pfizer and Roche; has participated in advisory boards for Exact Sciences, Gilead, Lilly, MSD, Pfizer, Roche, Seagen. BCSDI has received honoraria from Eisai, GSK and Novartis; travel grants from Novartis, Pfizer and Teva; has participated in advisory boards for Pfizer. MBE has received honoraria from Lilly, Novartis and Pfizer; travel grants from Pfizer; has participated in advisory boards for Lilly, Novartis and Pfizer. SDC and AG are Novartis employees. MM has received research grants from Novartis, PUMA and Roche; honoraria from Lilly, Novartis, Pierre Fabre, Pfizer and Roche; has a consulting or advisory role with AstraZeneca, Lilly, Novartis, Pharmamar, Pfizer, Roche and Taiho Pharmaceutical; has participated in speaker's bureaus for Lilly/ImClone, Pierre Fabre and Roche. CARS, BJR, NDF, NMJ, RDTS, and JIDM have no conflicts of interest to disclose.

## Ethics statement

The study was designed, implemented, and reported in accordance with the International Council for Harmonisation of Technical Requirements for Pharmaceuticals for Human Use Harmonized Tripartite Guidelines for Good Clinical Practice, with applicable local regulations, and with the ethical principles laid down in the Declaration of Helsinki. The protocol and informed consent form were reviewed and approved by a properly constituted Institutional Review Board/Independent Ethics Committee/Research Ethics Board before study commencement. Written informed consent was obtained from all patients. A steering committee oversaw the conduct of the trial as per the approved protocol. Representatives of the trial sponsor, Novartis Pharmaceuticals (East Hanover, NJ), collected and analyzed the data.

## Data sharing statement

Novartis is committed to sharing with qualified external researchers, access to patient-level data and supporting clinical documents from eligible studies. These requests are reviewed and approved by an independent review panel on the basis of scientific merit. All data provided is anonymized to respect the privacy of patients who have participated in the trial in line with applicable laws and regulations. This trial data availability is according to the criteria and process described on www.clinicalstudydatarequest.com.
