# Supplementary material for: Safety and efficacy of ribociclib plus letrozole in patients with HR+, HER2– advanced breast cancer: Results from the Spanish sub-population of the phase 3b CompLEEment-1 trial
Source: Breast. 2022 Sep 28;66:77–84. doi: 10.1016/j.breast.2022.09.006 (PMC9535465; doi:10.1016/j.breast.2022.09.006)
Supplement: Multimedia component 2 [file mmc2.docx]

**CONSORT diagram**

- Completed treatment: 221 (42.0%)
- Completed and entered Extension Phase: 1 (0.2%)

Discontinued from treatment: 305 (58.0%)

- Progressive disease: 178 (33.8%)
- Adverse event: 79 (15.0%)
- Physician decision: 17 (3.2%)
- Subject/guardian decision: 13 (2.5%)
- Protocol deviation: 8 (1.5%)
- Death: 7 (1.3%)
- Lost to follow-up: 2 (0.4%)
- Technical problems: 1 (0.2%)

Subgroup

n=526
