# Supplementary material for: Safety and efficacy of ribociclib plus letrozole in patients with HR+, HER2– advanced breast cancer: Results from the Spanish sub-population of the phase 3b CompLEEment-1 trial
Source: Breast. 2022 Sep 28;66:77–84. doi: 10.1016/j.breast.2022.09.006 (PMC9535465; doi:10.1016/j.breast.2022.09.006)
Supplement: Multimedia component 3 [file mmc3.docx]

**Supplementary Files**

**Supplementary Table 1.** Patient disposition.

| Disposition | Spanish Patients  n (%) |
| --- | --- |
| **Patients treated** | 526 (100.0) |
| **Completed treatment**  Completed and entered Extension Phase  Completed, did not enter Extension Phase | 221 (42.0)  1 (0.2)  220 (41.8) |
| **Discontinued from treatment** | 305 (58.0) |
| **Reason for discontinuation**  Progressive disease  Adverse event  Patient/guardian decision  Physician decision  Death  Protocol deviation  Lost to follow-up  Technical problems | 178 (33.8)  79 (15.0)  17 (3.2)  13 (2.5)  7 (1.3)  8 (1.5)  2 (0.4)  1 (0.2) |

**Supplementary Table 2.** Best overall response by subgroup (baseline patient characteristics) in patients with measurable disease at baseline.

| Best overall response^a^ | All patients^b^  N=298  n (%) | Age <50  N=98  n (%) | Age ≥50  N=200  n (%) | Premenopausal  N=99  n (%) | Postmenopausal  N=196  n (%) |
| --- | --- | --- | --- | --- | --- |
| CR  PR  SD  PD  UNK | 10 (3.4)  129 (43.3)  111 (37.2)  24 (8.1)  24 (8.1) | 5 (5.1)  49 (50.0)  30 (30.6)  11 (11.2)  3 (3.1) | 5 (2.5)  80 (40.0)  81 (40.5)  13 (6.5)  21 (10.5) | 5 (5.1)  41 (41.4)  35 (35.4)  11 (11.1)  7 (7.1) | 5 (2.6)  86 (43.9)  75 (38.3)  13 (6.6)  17 (8.7) |
| **ORR (95% CI)** | 46.6  (40.9, 52.5) | 55.1  (44.7, 65.2) | 42.5  (35.6, 49.7) | 46.5  (36.4, 56.8) | 46.4  (39.3, 53.7) |
| **CBR (95% CI)** | 68.5  (62.8, 73.7) | 74.5  (64.7, 82.8) | 65.5  (58.5, 72.1) | 68.7  (58.6, 77.6) | 68.4  (61.4, 74.8) |

^a^ As per local investigator's assessment for subgroups (Full Analysis Set). ^b^ Patients with measurable disease at baseline. CBR, clinical benefit rate; CI, confidence interval; CR, complete response; ORR, overall response rate; PD, progressive disease; PR, partial response; SD, stable disease; UNK, unknown.

**Supplementary Table 3.** Best overall response by subgroup (disease characteristics) in patients with measurable disease at baseline.

| Best overall response ^a^ | All patients^b^  N=298  n (%) | Visceral metastases  N=220  n (%) | No visceral metastases  N=78  n (%) | <3 metastatic sites  N=153  n (%) | ≥3 metastatic sites  N=145  n (%) | Prior chemotherapy  N=32  n (%) | No prior chemotherapy  N=266  n (%) |
| --- | --- | --- | --- | --- | --- | --- | --- |
| CR  PR  SD  PD  UNK | 10 (3.4)  129 (43.3)  111 (37.2)  24 (8.1)  24 (8.1) | 7 (3.2)  98 (44.5)  75 (34.1)  19 (8.6)  21 (9.5) | 3 (3.8)  31 (39.7)  36 (46.2)  5 (6.4)  3 (3.8) | 8 (5.2)  67 (43.8)  57 (37.3)  10 (6.5)  11 (7.2) | 2 (1.4)  62 (42.8)  54 (37.2)  14 (9.7)  13 (9.0) | 1 (3.1)  9 (28.1)  14 (43.8)  4 (12.5)  4 (12.5) | 9 (3.4)  120 (45.1)  97 (36.5)  20 (7.5)  20 (7.5) |
| **ORR (95% CI)** | 46.6  (40.9, 52.5) | 47.7  (41.0, 54.5) | 43.6  (32.4, 55.3) | 49.0  (40.9, 57.2) | 44.1  (35.9, 52.6) | 31.3  (16.1, 50.0) | 48.5  (42.3, 54.7) |
| **CBR (95% CI)** | 68.5  (62.8, 73.7) | 67.3  (60.6, 73.4) | 71.8  (60.5, 81.4) | 70.6  (62.7, 77.7) | 66.2  (57.9, 73.8) | 59.4  (40.6, 76.3) | 69.5  (63.6, 75.0) |

^a^ As per local investigator's assessment for subgroups (Full Analysis Set). ^b^ Patients with measurable disease at baseline. CBR, clinical benefit rate; CI, confidence interval; CR, complete response; ORR, overall response rate; PD, progressive disease; PR, partial response; SD, stable disease; UNK, unknown.

**Supplementary Figure 1.** Study design.


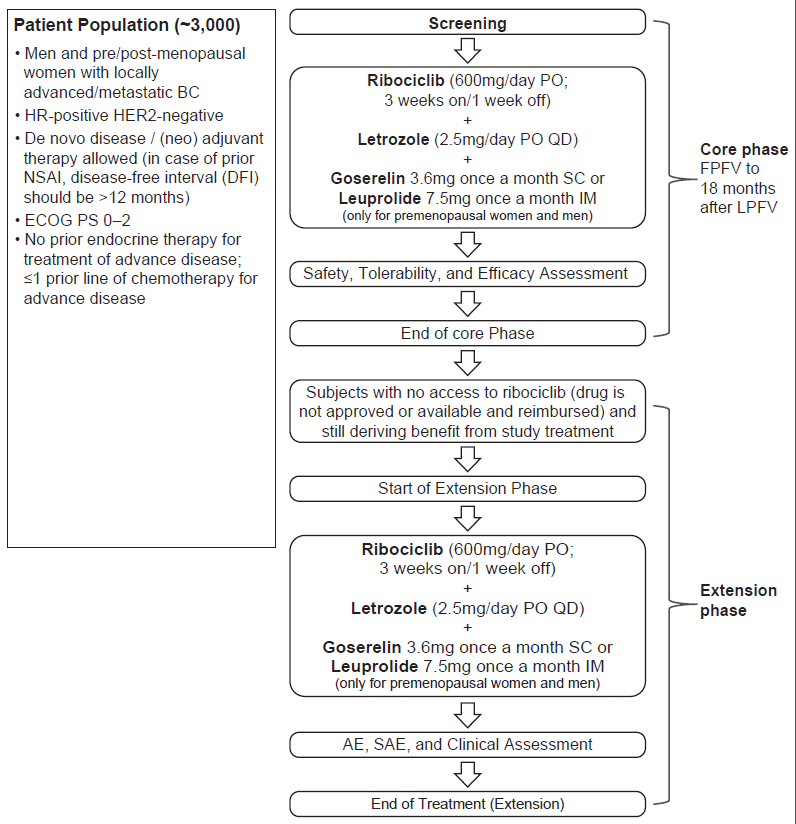


AE, adverse event; BC, breast cancer; ECOG PS, Eastern Cooperative Oncology Group performance status; FPFV, first patient first visit; HER2; human epidermal growth factor receptor 2; HR, hormone receptor; IM, intramuscular; LPFV, last patient first visit; NSAI, non-steroidal aromatase inhibitor; PO, oral; QD, once daily; SAE, serious adverse event; SC, subcutaneous.

**Supplementary Figure 2.** Patient-reported outcomes: Change from baseline in FACT-B scores.


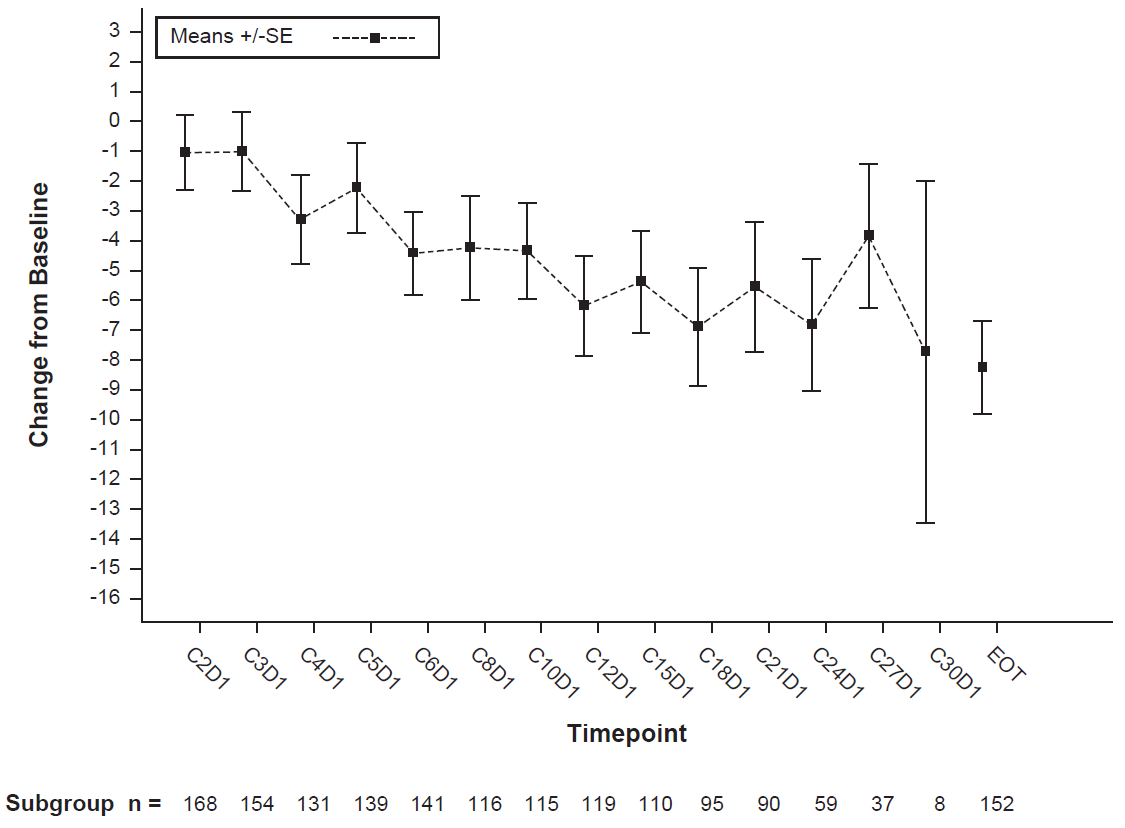


C, cycle; D, day; FACT-B, Functional Assessment of Cancer Therapy – Breast; SE, standard error.
